# Microbial recycling of plastics: An annotated selection of World Wide Web sites relevant to the topics in microbial biotechnology

**DOI:** 10.1111/1751-7915.14028

**Published:** 2022-03-10

**Authors:** Lawrence P. Wackett

**Affiliations:** ^1^ Department of Biochemistry Molecular Biology & Biophysics, BioTechnology Institute University of Minnesota St Paul MN 55108 USA

## Biodegradation of a plastic monomer

### https://www.manchester.ac.uk/discover/news/plastic‐eating‐bacteria‐could‐help‐aid‐global‐recycling‐efforts/

This news write‐up summarizes a paper describing the uptake and biodegradation of the plastic monomer compound terephthalic acid.

## Bacterial enzymes digest some plastic waste

### https://www.science.org/content/article/could‐plastic‐eating‐microbes‐take‐bite‐out‐recycling‐problem

This is an article in *Science* that provides a broad overview of developments in the breakdown and recycling of plastics by microbial enzymes.

## Business race for plastic‐eating bacteria

### https://www.forbes.com/sites/scottcarpenter/2021/03/10/the‐race‐to‐develop‐plastic‐eating‐bacteria/?sh=6b67decf7406

This article in the business publication *Forbes* discusses scientific and business aspects of finding, characterizing and commercializing bacteria that can degrade and recycle plastics.

## Review of plastics biodegradation

### https://www.frontiersin.org/articles/10.3389/fmicb.2020.00442/full

This paper provides a broad overview of biodegradation studies on synthetic plastics: polyethylene, polystyrene, polypropylene, polyvinyl chloride, polyurethane and polyethylene terephthalate.

## EU plastics recycling initiative

### https://enveurope.springeropen.com/articles/10.1186/s12302‐021‐00536‐5

This paper describes a large effort in Europe to use mixed bacteria and/or enzymes to degrade recalcitrant polymers and use them as feedstocks for microbial transformations.

## Plastics and microbiomes

### https://environmentalmicrobiome.biomedcentral.com/articles/10.1186/s40793‐020‐00371‐w

This article examines the effects and degradation of plastics in gut and environmental microbiomes.

## Plastic degradation potential by microbes

### https://journals.asm.org/doi/10.1128/mBio.02155‐21

This study developed HMMs of enzymes presumptively degrading plastics and used them in a global survey of biodegradation potential.

## Plastics to products

### https://www.sciencedaily.com/releases/2021/06/210610135752.htm

This news‐type report describes a study conducted to biodegrade the polymer polyethylene terephthalate and use the aromatic monomer to make the food additive vanillin by using a recombinant bacterium.

## X‐ray structure of PETase

### https://www.rcsb.org/structure/6EQH

This is a link to the X‐ray structure of a polyethylene terephthalate degrading hydrolase from the bacterium *Ideonella sakaiensis*.

## PMBD: Plastics biodegradation database

### http://pmbd.genome‐mining.cn/home/

This database focuses on microbial genes involved in plastic biodegradation.

## Plastic‐degrading bacteria

### https://msystems.asm.org/content/6/1/e01112‐20

This study looked at the phylogenetics of plastic‐degrading bacteria, seeking to link types of bacteria with specific plastics.

